# Apaf1 inhibition promotes cell recovery from apoptosis

**DOI:** 10.1007/s13238-015-0200-2

**Published:** 2015-09-11

**Authors:** Anna Gortat, Mónica Sancho, Laura Mondragón, Àngel Messeguer, Enrique Pérez-Payá, Mar Orzáez

**Affiliations:** Laboratory of Peptide and Protein Chemistry, Centro de Investigación Príncipe Felipe, 46012 Valencia, Spain; Department of Chemical and Biomolecular Nanotechnology, Instituto Química Avanzada de Cataluña (CSIC), 08034 Barcelona, Spain; Instituto de Biomedicina de Valencia, IBV-CSIC, 46010 Valencia, Spain

**Keywords:** Apaf1, Apaf1 inhibitors, apoptosis, apoptosome, autophagy, cell recovery

## Abstract

The protein apoptotic protease activating factor 1 (Apaf1) is the central component of the apoptosome, a multiprotein complex that activates procaspase-9 after cytochrome *c* release from the mitochondria in the intrinsic pathway of apoptosis. We have developed a vital method that allows fluorescence-activated cell sorting of cells at different stages of the apoptotic pathway and demonstrated that upon pharmacological inhibition of Apaf1, cells recover from doxorubicin- or hypoxia-induced early apoptosis to normal healthy cell. Inhibiting Apaf1 not only prevents procaspase-9 activation but delays massive mitochondrial damage allowing cell recovery.

## INTRODUCTION

Apoptosis is executed by highly regulated cellular pathways and is crucial for the removal of cells with damaged DNA or organelles, viral or bacterial infections as well as superfluous or ectopic cells. Two major signaling pathways have been described for apoptosis induction. The extrinsic pathway is initiated through external stress signals that induce the binding of plasma membrane proteins of the death receptor family (Peter and Krammer, 2003). The intrinsic pathway can be initiated by a number of factors including growth factor withdrawal and genotoxic stress which, with the participation of members of the Bcl-2 family of proteins, lead to mitochondrial outer membrane permeabilization (MOMP) (Brenner and Mak, 2009). This is followed by cytochrome *c* (Cyt *c*) release from mitochondria and formation of the cytosolic multiprotein complex termed the apoptosome. Assembly of the apoptosome triggers signaling cascades that activate the caspase family of cysteine aspartyl proteases. These caspases are essential to the initiation and execution of the apoptotic process. Apoptosis dysregulation is at the root of a variety of diseases. Apoptosis resistance has been causally linked to cancer and autoimmune diseases, and conversely, excessive apoptosis promotes pathological conditions related to stroke, ischemia-reperfusion damage and degenerative diseases (Green and Kroemer, 2005). While the development of new anti-cancer therapies relies on inducing apoptosis (Debatin, 2006), progress has been much slower in the discovery of inhibitors of unwanted apoptosis. Initial drug discovery efforts were directed towards the identification of executioner caspase-3 inhibitors (Linton, 2005). The caspase-3 inhibitors showed promising potential in several animal models of alcoholic liver diseases, hepatitis B and C virus infection (Kunstle et al., 1997), myocardial infarction and infarct size in stroke (Cheng et al., 1998; Yaoita et al., 2000), sepsis (Hotchkiss and Karl, 2003), neuronal death and in spinal cord injury (Springer et al., 1999). However, caspase inhibitor-based drugs are experimenting slow pharmacological advance due to the reported side effects especially in terms of hepatotoxicity (Schotte et al., 1999; Van Noorden, 2001; Hoglen et al., 2004). In addition, in the development of apoptosis inhibitors it should be considered, the necessity of maintaining cell functionality in order to provide long term survival rather than simply inhibiting death. Some lines of evidence showed that merely executioner caspase inhibition would not reach this objective. In a model of Parkinson’s disease it was reported successful inhibition of neuronal cells death upon treatment with Z-Val-Ala-Asp(OMe)-fluoromethylketone (zVAD-fmk), an irreversible caspase inhibitor; however, the loss of neurite or reduction of dopamine uptake was not improved with this treatment (von Coelln et al., 2001).

When reviewing the apoptosis signaling pathway there are several points susceptible of intervention to develop unwanted cell death inhibitors. In this sense, the formation of the apoptosome offered evidences to be considered as an interesting target as it is downstream from the mitochondrial events that characterize the pathway but upstream of effector caspases (e.g., caspase-3/7) that are the executors of cell death. The main constituent of the apoptosome is Apaf1 a multidomain protein that activates upon binding to Cyt *c* released from mitochondria after MOMP (Srinivasula et al., 1998). Mochizuki et al. (2001) reported that an adeno-associate virus vector-based delivery of an Apaf1 dominant negative inhibitor was able to inhibit apoptosis-mediated degeneration of nigrostriatal neurons in a MPTP model of Parkinson’s disease. Gao et al. (2009), by means of the overexpression of the specific Apaf1 inhibitory protein (AIP), have reported that inhibition of Apaf1 in an animal model of neonatal hypoxic-ischemic brain injury resulted in an attenuated brain tissue loss. In Apaf1 deficient cells, Ferraro et al. have demonstrated that such cells can turn-on readjustments of metabolic pathways to survive apoptotic stimulus while the depolarized state of mitochondria is reverted (Ferraro et al., 2008). Small molecules that inhibit Apaf1 are another promising approach for developing unwanted apoptosis inhibitors. We have reported on a family of small molecules that inhibits apoptosis by interfering with the apoptosome activity (Malet et al., 2006; Mondragon et al., 2008; Mondragon et al., 2009; Santamaria et al., 2009; Orzaez et al., 2014; Sancho et al., 2014b). In particular, SVT016426 was as efficient as the caspase inhibitor zVAD-fmk inhibiting the intrinsic apoptotic pathway. Here we show that the apoptosis inhibition provided by the Apaf1 inhibitor SVT016426 at the level of apoptosome contributes to maintain functional cells, thus raising hope for the development of future treatments of unwanted pathological apoptosis. Understanding the physiology of cell death has allowed the development of mechanistic approaches for the development of apoptosis-related drugs. However to properly face death prevention and most importantly cell recovery from early apoptosis stages, we have to understand not only how cells die but also how cells recover. We report here on a method to distinguish and to classify living cells at different stages of apoptosis. The possibility of isolating cells at an early apoptotic phase allowed us to identify autophagy as the molecular mechanism that facilitates SVT016426-dependent cell recovery.

## RESULTS

### Apaf1 inhibition provides survival to cells induced to execute apoptosis

**Figure 1:**
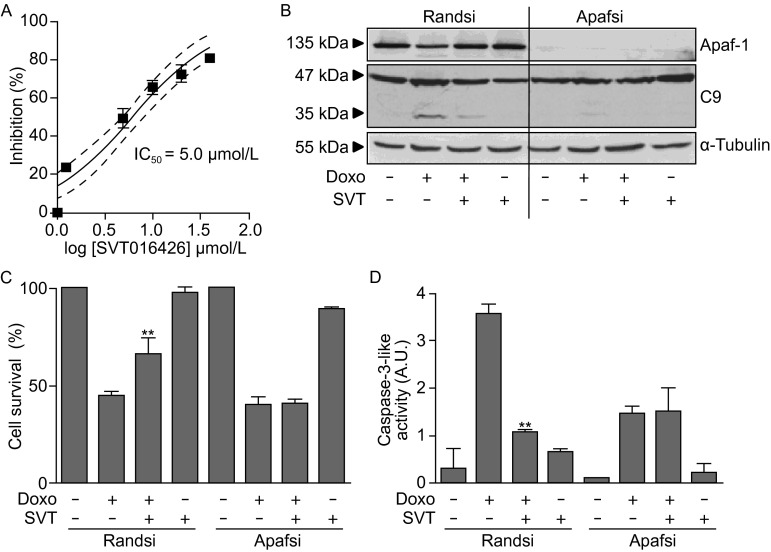
**Chemical inhibition of Apaf1 provides cell survival**. (A) The IC_50_ of SVT016426 was analyzed using HeLa S100 cell extract-based assay. Caspase-3-like activity on HeLa S100 extracts was measured in the presence or absence of increasing concentrations of SVT016426. Data are presented as mean inhibition percentage of control ± SD, *n* = 3. Curve fitting was performed using GraphPad Prism 3.0 software. (B) Apaf1 was silenced in HeLa cells by siRNA (100 nmol/L) methodology. Immunoblotting for Apaf1, caspase-9 and α-tubulin (loading control) was provided in cell extracts silenced using a random siRNA (Randsi) or Apaf1 siRNA (Apafsi). Cell survival (C) and caspase-3-like activity (D) were measured for control (Randsi) or Apaf1 (Apafsi) silencing in the presence or absence of Doxo (1 µmol/L) and SVT016426 treatment (SVT; 5 µmol/L) (mean ± SD, *n* = 3). Cell viability was measured by both, trypan blue and MTT assay. Asterisks represent significant differences relative to apoptotic treatment as determined by one-way ANOVA test with Bonferroni’s multiple comparison post-test (**P < 0.05)

Direct damage to cells causes individual cell death that depending on the number of cell loss can result on tissue or organ failure; e.g. cardiac damage that occurs late after chemotherapy (months or even a year or more) is one of the major side effects of doxorubicin (Doxo) treatment, a drug that is one of the most widely used anticancer drugs for solid tumors (Takemura and Fujiwara, 2007). In other cases, as stroke or tissue infarction, a hypoperfused, hypoxic, meta-stable region, named the penumbra, is formed around the core of necrotic cell death. The penumbra region retains structural integrity but has a compromised functionality and its long term recovery defines the basis for stroke and/or tissue infarction therapy (Yuan, 2009). We asked whether Apaf1 inhibition by SVT016426 could have application in hypoxia and Doxo-induced cell death. Chemical inhibitors of Apaf1, as SVT016426, inhibit the apoptosome-dependent induction phase in different cells induced to execute apoptosis (Malet et al., 2006; Mondragon et al., 2008; Mondragon et al., 2009; Orzaez et al., 2014). Then, we initially analyzed the ability of SVT016426 to inhibit apoptosome activity in HeLa cell extracts. Incubation of the cytosolic S100 cell extract with dATP and Cyt *c* restored the apoptotic pathway through induction of the apoptosome formation (Fearnhead, 2001); this restoration was followed using a fluorogenic substrate for caspases (Ac-DEVD-afc). SVT016426 treatment inhibited Apaf1-induced activation of caspase activity (Fig. 1A). We also analyzed target-specificity of SVT016426 in a model of Doxo-induced apoptosis in HeLa cells. For this purpose, we considered the use of small interfering RNA (siRNA)-based silencing of Apaf1 (Fig. 1B) and analyzed the activity of SVT016426 in Doxo-induced cell death in the presence or absence of Apaf1 in the cells. When HeLa cells transfected with a control random siRNA were treated with Doxo we obtained close to 60% of Doxo-induced cell death. However in the presence of SVT016426 death decreased to a 40% of the cell population (Fig. 1C). In contrast, Doxo-induced cell death was not inhibited by SVT016426 in Apaf1 siRNA-based knockdown cells (Fig. 1C). It should be mentioned here that in the absence of Apaf1, Doxo induced a caspase-independent cell death in these cells as it was described previously (Miyazaki et al., 2001; Andreu-Fernandez et al., 2013; Sancho et al., 2014a). These cell viability results were well correlated with caspase-9 processing (Fig. 1B) and measurements of caspase-3 activity (Fig. 1D) suggesting that SVT016426 inhibitory capacity was dependent on the levels of Apaf1 in the cell. These observations imply that SVT016426-mediated inhibition of Apaf1 results in pathway responses and cellular phenotypic effects compatible with an Apaf1-selective inhibition of apoptosis. Then SVT016426 not only inhibited caspase activity but also inhibited cell death. The SVT016426-induced cell death inhibition was close to 20% of the total cell population. This death inhibition ratio, although still modest, could be of relevance finding ways to describe future apoptosis inhibition-based therapies to devastating diseases as neurodegenerative diseases and to decrease secondary effects of cancer chemotherapy.

### Apaf1 inhibition permits cell recovery after hypoxia or DNA-damage

We were interested in analyzing not only cell death inhibition but also the possibility of SVT016426-dependent cell recovery. As SVT016426 acts downstream mitochondria we reasoned that only cells in the early phases of apoptosis would be able to recover.

**Figure 2:**
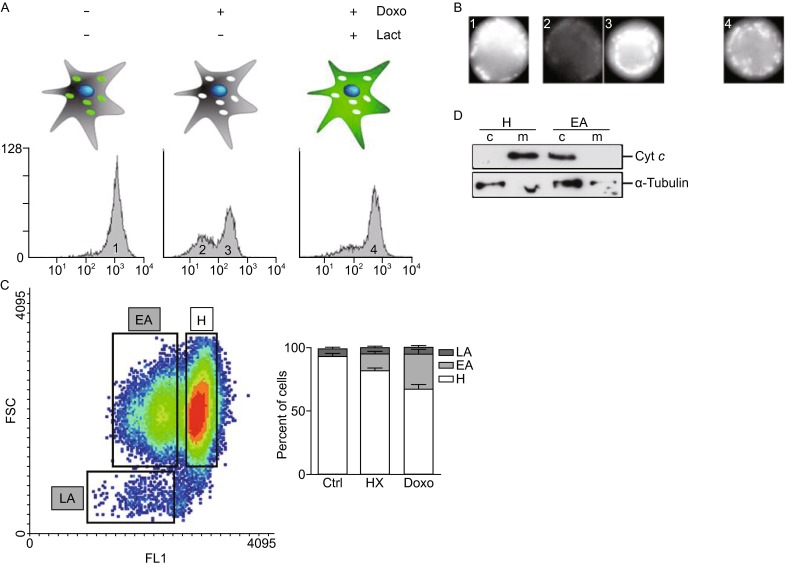
**An experimental model to separate cellular populations at different stages of apoptotic cell death based on Cyt** ***c*** **localization**. (A) Determination of Cyt *c* release in HeLa-GFPst by flow cytometry. HeLa-GFPst cells were induced to apoptosis with 1 µmol/L Doxo for 6 h in the presence or absence of 10 µmol/L of the proteasome inhibitor lactacystin. Samples were subjected to cytofluorometric analysis to detect Cyt *c*-GFP. Two peaks of different intensity can be distinguished. The high intensity peak corresponds to healthy cells (Cyt *c* in the mitochondria). After an apoptotic insult a second peak of low intensity appears (Cyt *c* released and degraded by the proteasome in the cytosol). Inhibition of the proteasome under an apoptotic insult recovers the high intensity peak probing that the degradation of Cyt *c* in the cytoplasm is responsible of the low intensity peak. (B) HeLa-GFP cells treated as described in (A) were submitted to cell sorting. Representative fluorescence microscopy images of the different cellular populations corresponding to peaks in (A) are shown. (C) HeLa-GFPst cells were induced to apoptosis either submitting cells to HX for 4 days or adding 0.25 µmol/L Doxo for 24 h. Three different populations corresponding to healthy (H), early apoptotic (EA) and late apoptotic (LA) could be identified according to the degree of Cyt *c* release and cellular size. Quantification of the different populations observed in the absence (Ctrl) or in the presence of the apoptotic insults (HX; Doxo) are shown in the right panel. (D) Endogenous Cyt *c* localized to mitochondrial (m) and cytosolic (c) fractions in H and EA populations, respectively

To test this hypothesis we designed a cellular assay to separate populations in different apoptotic stages. We enriched the early apoptotic cell population by applying soft conditions of apoptosis induction. Then we developed a method based in HeLa cells that overexpressed Cyt *c*-GFP cells (HeLa-GFP) that allowed the analysis of cellular Cyt *c*-GFP distribution in living cells. However, we found that due to overexpression, in homeostatic HeLa-GFP cells a fraction of Cyt *c*-GFP was located in the cytoplasm. Then, HeLa-GFP cells were subjected to fluorescence-activated cell sorting (named thereof sorting, st) to select a specific and homogeneous HeLa-GFPst cell population with moderate Cyt *c*-GFP expression levels and absence of Cyt *c*-GFP in cytosol to allow further quantitative procedures. This HeLa-GFPst population allowed setting up a method to analyze Cyt *c* release in living cells subjected to long hypoxia conditions and to sub-lethal Doxo treatment. Cyt *c* released from mitochondria upon an apoptotic stimuli, is rapidly degraded by the proteasome (MacFarlane et al., 2002; Rehm et al., 2003; Ferraro et al., 2008), then Cyt *c*-GFP released from HeLa-GFPst mitochondria will produce a change in fluorescence signal intensity allowing cytofluorimetric measurements without necessity for cell permeabilization. This vital method would allow the cell sorting procedures required for the analysis of cells at different stages of the apoptotic pathway (see below). We initially set-up the method with Doxo-induced apoptotic HeLa-GFPst cells submitted to cytofluorimetric measurements using the 520 nm channel to detect GFP fluorescence. We observed the appearance of a GFP low-intensity peak (Fig. 2A and 2B; peak 2) upon apoptosis induction which was very significantly reduced by lactacystin-induced proteasome inhibition, thus validating the set-up (Fig. 2A and 2B; peak 4) (Rehm et al., 2003). To quantitatively analyze the progression of cells through the cell death pathway according to the cytofluorimetric results, three independent cell populations were defined according to their GFP fluorescence signal intensity and size (Fig. 2C) upon apoptotic induction. The cell population with an average size characteristic of the HeLa-GFPst cell line (as assessed by forward scatter - FS+) and high GFP fluorescence was considered as healthy (H; GFP+/FS+). Cells showing FS+ and diminished GFP fluorescence signal were considered as early apoptotic (EA; GFP-/FS+) and the cell population of reduced size (as assessed by forward scatter - FS-) and low GFP fluorescent signal was considered as late apoptotic (LA; GFP-/FS-). According to such cell population definition, we applied sorting to obtain differentially purified cell populations that were analyzed for GFP signal under fluorescence microscope (Fig. 2B) and for endogenous Cyt *c* subcellular localization upon cell fractionation (Fig. 2D) to corroborate that our measurements of Cyt *c*-GFP mirrored the general behavior of endogenous Cyt *c*. As expected, H cells appeared intensely stained with GFP bearing a clear hallmark of mitochondrial network in both, untreated population (Fig. 2B, peak 1) as well as in cells isolated from the GFP+/FS+ peak upon Doxo treatment (Fig. 2B, peak 3). In contrast, cells pooled from the GFP- peak that appeared upon Doxo treatment were significantly less intense (Fig. 2B, peak 2) whereas GFP-associated fluorescence intensity of Doxo and lactacystin-treated cells was similar to that of the healthy untreated cells (Fig. 2B, peak 4). Importantly, endogenous Cyt *c* was confined to mitochondria in H cell population while it was found at cytosolic fractions in EA cell population (Fig. 2D). These results suggest that mitochondrial released Cyt *c* is susceptible of a proteasome-dependent degradation that can be partially inhibited by proteasome inhibitors. To further validate our experimental system of purification of cell populations at different stages in the apoptotic pathway, we performed measurements of caspase-3/7 activity. As expected, no caspase-3/7 activity was detected in H or EA cells (results not shown).

**Figure 3:**
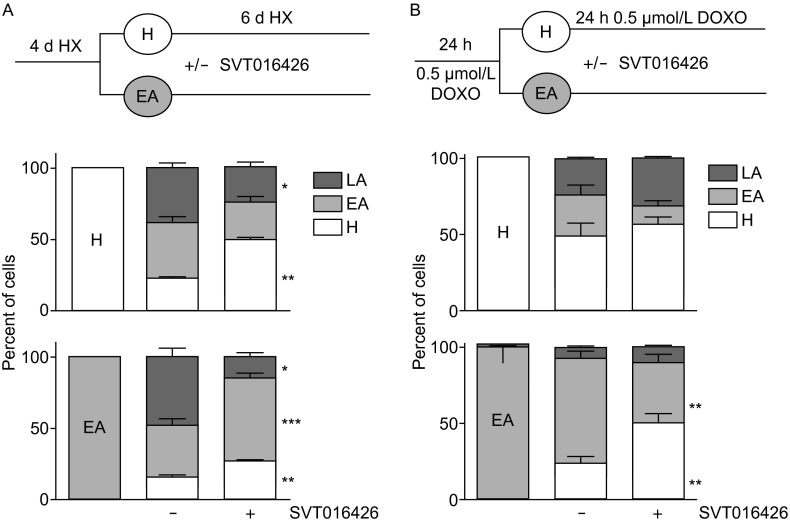
**Apaf1 chemical inhibition allows cell recovery after the onset of apoptosis**. (A) HeLa-GFPst cells were sorted after 4 days of HX into H, EA and LA (not shown) populations. H and EA cells were reseeded and resubmitted to HX for 6 additional days in the presence or absence of 5 µmol/L SVT016426. The evolution of the different populations at the end of the experiment was quantified by flow cytometry. (B) HeLa-GFPst cells were induced to apoptosis by adding 0.25 µmol/L Doxo. As in (A), cells were sorted into H, EA and LA (not shown) populations. H and EA cells were reseeded and treated with 0.5 µmol/L Doxo for an additional period of 24 h in the presence or absence of 5 µmol/L SVT016426. The evolution of the different populations at the end of the experiment was quantified by flow cytometry. Data represent mean ± SD of three independent experiments

Next, we addressed the question if apoptosis induced by different apoptotic insults can be not only prevented by SVT016426-dependent Apaf1 inhibition but also whether or not cells can be recovered from such insults. Experimental cellular models of hypoxia and Doxo-induced apoptosis were then evaluated. HeLa-GFPst cells were incubated in hypoxia/hypercapnea (HX) conditions (1% O_2_, 18% CO_2_) conditions for 4 days or treated with 0.25 µmol/L Doxo for 24 h and submitted to sorting in order to obtain clean pools of H, EA and LA cells. The cross-contamination among the different pools was thoroughly controlled and was kept as low as less than 1% (0.6%–0.7% in most cases, data not shown). The three different cell pools were then plated on dishes and maintained in HX conditions for 6 additional days or treated further with 0.5 µmol/L Doxo for 24 h in the presence or absence of 5 µmol/L SVT016426 (scheme in Fig. 3A and 3B upper panels). The cytofluorimetric measurements revealed that only 30% of the initial H cells in prolonged HX conditions and 50% of the Doxo-treated H cells remained without entering the cell death pathway. However, in the presence of the Apaf1 inhibitor SVT016426 the number of unaffected H cells increased to 49% ± 2% and to 56% ± 5% for the HX and Doxo treated cells, respectively. This increase was correlated with a 1.6-fold reduction of the number of LA cells (from 38% ± 4% to 23% ± 4%) in the case of the HX population, thus confirming the protective effect that chemical Apaf1 inhibitors exert on cells previous to the apoptotic insult. The EA pool subjected to the HX conditions by the 6 additional days or exposed to Doxo progressed through the cell death pathway as assessed by the presence of 48% ± 7% or 7% ± 1% of LA cells, respectively (Fig. 3A and 3B lower panels; Fig. S1B). In HX conditions, treatment with SVT016426 decreased the number of the LA cells by a 3.3-fold to 15% ± 2% concomitant with a markedly increase in the EA population (37% ± 4% to 58% ± 4% in control and SVT016426 treated, respectively). Moreover, we observed that the presence of H cells from a pool of pure EA cells was substantially increased in those EA cells treated with SVT016426 (from 15% ± 2% to 27% ± 1% on the EA cell population under HX and from 23% ± 5% to 50% ± 6% in the EA cell population treated with Doxo) (Fig. 3A and 3B lower panel white bars; Fig. S1A). These results suggest that cells which were plated as a pure EA population triggered an SVT016426-dependent recovery program. Then, inhibition of Apaf1 not only prevents cells entering the apoptotic program but helps cells to recover from early apoptotic stages. SVT016426 treated cells are able to initiate a normal cell division program as previously demonstrated in colony formation assays (Orzaez et al., 2014). As expected, the LA population did not undergo any changes (data not shown).

### Autophagy is implicated in SVT016426 cell recovery

**Figure 4:**
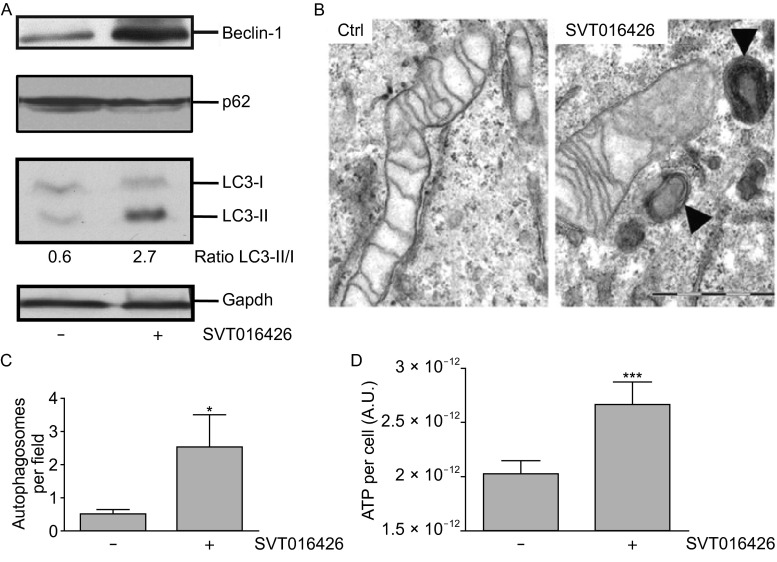
**Autophagy is implicated in cell recovery upon Apaf1 inhibition**. (A) HeLa cells were treated as in Fig. 3A and expression changes of proteins implicated in autophagy, namely Beclin-1, p62 and LC3-I and -II (the lipidated form of LC3-I), were evaluated by Western blot. Gapdh was used as loading control. (B) Cellular morphology of HX-treated HeLa cells was analyzed by transmission electron microscopy (TEM). Representative images of HX-control and HX-SVT016426 treated cells are shown (Black arrows indicate the presence of autophagosomes). Scale bar equal to 1 µm. (C) Quantification of the number of autophagosomes per field was performed analyzing 40 randomly selected fields per image from two independent experiments. (D) The ATP content per cell in the presence or absence of 5 µmol/L SVT016426 was measured. Results shown are the mean of three independent experiments. Statistical significance was assessed by means of two-tailed Student’s *t*-test (*P < 0.05; ***P < 0.001)

**Figure 5:**
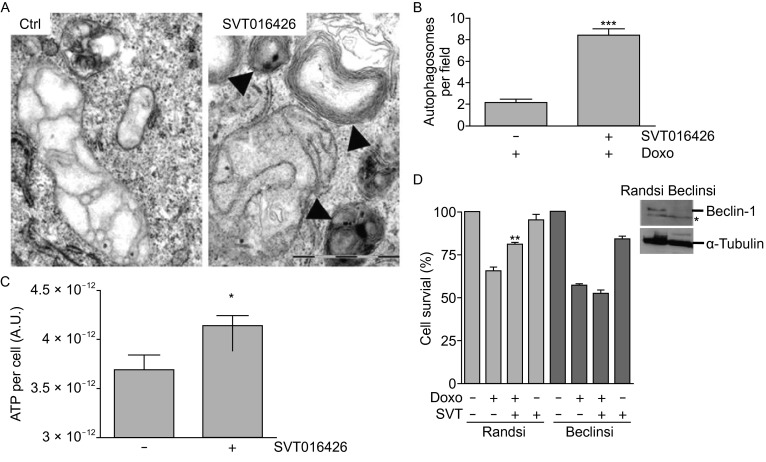
**Autophagy is implicated in cell recovery upon Apaf1 inhibition**. (A) Representative TEM images of HeLa cells treated with 0.25 µmol/L Doxo for 24 h and maintained for additional 24 h with 0.5 µmol/L Doxo in the presence or absence of 5 µmol/L SVT016426. Black arrows indicate the presence of autophagosomes. Scale bar equal to 1 µm. (B) Quantification of the number of autophagosomes per field was performed analyzing 40 randomly selected fields per image from two independent experiments. (C) Measurement of ATP content per cell in HeLa cells treated as described in (A) in the presence or absence of 5 µmol/L SVT016426. Statistical significance was assessed by means of two-tailed Student’s *t*-test (*P < 0.05; ***P < 0.001). (D) Cell survival was measured by MTT for control (Randsi) or Beclin (Beclinsi) silencing in the presence or absence of Doxo (1 µmol/L) and SVT016426 treatment (SVT; 5 µmol/L) (mean ± SD, *n* = 3). Asterisks represent significant differences relative to apoptotic treatment as determined by one-way ANOVA test with Bonferroni’s multiple comparison post-test (**P < 0.05). Immunoblotting for Beclin-1 and α-tubulin (loading control) was provided in cell extracts silenced using a random siRNA (Randsi) or Beclin siRNA (Beclinsi). Asterisk marks for unspecific bands recognized by the antibody

Autophagy is a catabolic cytoprotective mechanism that constitutes a cellular defense against stress induced by starvation (Madeo et al., 2010). It is characterized by the formation of double-membrane vesicles named autophagosomes that engulf cytoplasmic organelles and proteins. The autophagosomes fuse with lysosomes and their luminal content is degraded. Beclin-1 is a protein characterized as essential for the initiation of autophagy. In fact, it has been demonstrated that is the activation of a Beclin-1 dependent autophagy pathway that permits glycolytic-dependent ATP generation allowing recovery from death-stimulus of apoptosome-deficient proneural cells (Ferraro et al., 2008). We were interested in analyzing the role of autophagy in the SVT016426-dependent cell recovery from an early apoptotic to a healthy state. Thus, we analyzed the protein levels of Beclin-1, p62 and the lipidated form of the microtubule-associated protein 1A/1B-light chain 3 alpha type I (LC3-I) (Kabeya et al., 2000; Kabeya et al., 2004) as hallmarks of induced autophagy. Indeed, we observed an overexpression of Beclin-1, a lipidation of the LC3-I (LC3-II) and, a moderate decrease of p62 substrate, in SVT016426-treated when compared to unprotected HeLa cells under HX conditions (Fig. 4A), thus suggesting an increased rate of autophagy (Boya et al., 2005; Pattingre et al., 2005). The presence of autophagosome structures was also observed by transmission electron microscopy (TEM, Fig. 4B and 4C). Similar observations were derived from SVT016426-treated cells when Doxo was used as apoptotic insult (Fig. 5A and 5B). The SVT016426-dependent autophagy induction correlated with an increment in the ATP levels in SVT016426-treated HeLa cells under HX conditions (Fig. 4D) or treated with Doxo (Fig. 5C) when compared with cells not receiving the SVT016426 treatment. Finally, to confirm that cell recovery was mediated by the activation of the autophagy program we silenced Beclin-1 in the Doxo model and cell survival was analyzed. As expected, recovery by SVT016426 is not observed when Beclin-1 is knocked-down thus reinforcing the hypothesis that autophagy could be implicated in cell recovery (Fig. 5D).

## DISCUSSION

Here we have described an Apaf1-dependent cell program that permits cells to recover from intrinsic apoptotic insults. The use of selective chemical Apaf1 inhibitors (SVT016426 is a representative member) as chemical biology probes highlighted Apaf1 as a principal regulator not only in cell death but also in cell recovery pathways. As shown here, chemical inhibition of Apaf1 is necessary to allow intrinsic apoptosis-injured cells to have access to survival events that implies the participation of autophagy. The pro-survival adaptive function of autophagy acquires then relevance in this context. Our results suggest that cells at early stages of execution of the apoptotic program, even after Cyt *c* loss, can be recovered to cells with hallmarks of normal (healthy) cells. The cells readapt metabolic conditions and activate autophagy to sustain ATP production. It has been previously shown that Apaf1 genetically deprived proneural cells of embryonic origin can recover from apoptosis when glycolysis is active and the apoptotic stimulus is removed (Ferraro et al., 2008).

Our study combines the use of homogeneous well defined cell populations at different stages of apoptosis with small molecules that allowed the characterization of specific recovery programs. In cells of mammalian origin it has been long observed that the responsiveness to physiological stimuli is not uniform. In particular when a clonal cell population is induced to commit apoptosis some cells die while others survive. Among cells that survive one can probably find cells at different stages of apoptosis as it has been reported when extrinsic apoptosis was induced (Albeck et al., 2008). In this study we have validated a flow cytometry-based method to distinguish and to split the different cell populations originated when an original cell population was subjected to intrinsic apoptosis stimuli. The efficient characterization of three (healthy, early- and late-apoptosis) populations has permitted to unambiguously determine that the Apaf1 inhibitor not only prevents cell death but helps cells to recover from apoptosis. Overall, SVT016426 provides a powerful tool for characterizing the role of Apaf1. Such inhibitors were originally identified while searching for compounds that could prevent the activation of procaspase-9 when analyzed in an *in vitro* reconstituted apoptosome (Malet et al., 2006). It is now well established that the Apaf1 inhibitors provide cytoprotective effect to cells subjected to apoptosis stimulus in different cellular models (Malet et al., 2006; Mondragon et al., 2008; Santamaria et al., 2009; Orzaez et al., 2014; Sancho et al., 2014b). Furthermore, the Apaf1 inhibitor has been used to confirm the effect of caspase-3 inhibition on GluR1 synaptic distribution in a mouse model of Alzheimer’s disease (D’Amelio et al., 2011).

The discovery of candidate drugs that attenuate the effects of unwanted excessive apoptosis will benefit from the knowledge of how after an apoptotic stimulus, cells are able to recover and the molecular mechanisms that use cells to this end. Recent data (Ferraro et al., 2008) suggest that such cellular recovery should use still undiscovered pathways that link intrinsic apoptosis and metabolism networks. Albeck et al. (Albeck et al., 2008) using single-cell analysis approaches demonstrated that upon defined metabolic conditions, cells induced to extrinsic apoptosis can recover. In contrast, the links between the intrinsic apoptosis pathway and cellular pathways that could permit cell recovery still present more questions than answers. Nevertheless current knowledge suggests that the apoptotic machinery should interact functionally with key decision-making components of cellular metabolism (Colell et al., 2007; Ferraro et al., 2008) cell growth and cell division. These components would provide crosstalks between the intricate cellular pathways that define a particular cellular fate.

Early studies pointed to inhibitors of caspases as the most attractive molecules to develop anti-apoptotic drugs; however, chemical inhibition or knocking-out caspases is only able to prevent apoptosis but not cell death (Boya et al., 2005). In these conditions cells die by different processes. Using our system to split cells at different stages of the apoptotic program, we demonstrated that inhibition of Apaf1 enhanced apparition of healthy cells within the early apoptotic population. Similar behavior has been reported in *C. elegans*. Cells that undergo morphological changes accompanying CED-3 activation could recover completely when subjected to weak pro-apoptotic signals if they were not engulfed by phagocytic cells (Hoeppner et al., 2001; Reddien et al., 2001).

A cell full recovery mechanism must be a complex process that has to overcome irreversible caspase-dependent cleavage of substrates through restraining caspase-3 catalytic activity. This function is played by inhibitor of apoptosis proteins (IAPs) and proteasome-dependent caspase degradation (Albeck et al., 2008). Our data identify Apaf1 as a molecule that should be included in light of its role, when chemically inhibited, as a molecule participating in the process.

Several evidences have demonstrated that differentiated cells can recover after MOMP (Martinou et al., 1999; Deshmukh et al., 2000; Tang et al., 2009) and our study identifies Apaf1 and autophagy induction as part of the molecular mechanism of the recovery process, in particular in cells affected by hypoxic or sublethal doses of intrinsic apoptosis inducers. However, which is the mechanism and the components of the machinery providing cell recovery and whether and how they regulate apoptosis remain to be elucidated.

## MATERIALS AND METHODS

### Cell culture, reagents and antibodies

HeLa and HeLa-GFP cells were maintained in Dulbecco’s Modified Eagle’s Medium (DMEM) supplemented with 10% foetal bovine serum (FBS). Media and supplements for cell culture were purchased from Gibco-Invitrogen. Doxorrubicin (Doxo) was obtained from Sigma-Aldrich. Anti LC3 and anti caspase-9 antibodies were purchased from Cell Signaling. Anti Beclin-1 and anti Apaf1 antibodies were purchased from BD Transduction Laboratories, p62 antibody from Enzo Life Sciences and anti α-tubulin from Sigma-Aldrich.

### Cell-free caspase activation assay

S100 cytosolic extract from HeLa was obtained as previously described (Dignam et al., 1983; Malet et al., 2006). Extract (0.1 mg/mL) was incubated with different concentrations of SVT016426 (0, 1, 5, 10 and 40 µmol/L) in assay buffer (20 mmol/L HEPES, 10 mmol/L KCl, 1.5 mmol/L MgCl_2_, 1 mmol/L EDTA, 1 mmol/L EGTA, 1 mmol/L DTT, 0.1 mmol/L PMSF) plus dATP (10 mmol/L) and Cyt *c* (10 µmol/L) for 40 min at 30°C. Ac-DEVD-afc substrate (20 μmol/L; Enzo Life Sciences) was used to measure caspase-3-like activity using a Victor 2 spectrofluorimeter.

### Cell-based caspase activation assay

HeLa cells were seeded in 6-well plate at a cellular density of 1 × 10^5^ cells/mL. Then, Lipofectamine^TM^ 2000 (Invitrogen) was used according to the manufacturer’s instructions to transfect cells with a control random siRNA (Randsi; 100 nmol/L) and Apaf1 siRNA (Apafsi; 100 nmol/L) from Cell Signaling. 24 h later, cells were pre-treated with SVT016426 (5 µmol/L) for 1 h, then Doxo (1 µmol/L) was added. After 24 h cells were harvested and cytosolic extracts were obtained as described previously. Total protein (50 µg) was mixed with assay buffer containing 20 μmol/L Ac-DEVD-afc substrate. Activity was measured using a Victor 2 spectrofluorimeter.

### Trypan blue cell viability assay

HeLa cells seeded and treated as described before, were detached and 0.5% trypan blue dye added in solution. Live cells possess intact cell membranes that exclude the dye, whereas dead cells do not. Unstained (viable) and stained (non-viable) cells were counted separately in a hemacytometer and total number of viable cells in the population was calculated.

### MTT assay

Mitochondrial activity was measured using a MTT colorimetric assay. HeLa cells were grown in 24-well plates at a cell density of 1.0 × 10^5^ cells/well. After 24 h, cells were transfected with a control random siRNA (Randsi; 100 nmol/L) and Beclin-1 siRNA (Beclinsi; 100 nmol/L) from Cell Signaling. 24 h later, cells were treated with SVT016426 (5 µmol/L) and Doxo (1 µmol/L) for 30 h. Four hours before the end of the treatment 20 µL/well of MTT (5 mg/mL in PBS) was added to each well and the plates were incubated for a further 4 h at 37°C. Finally, the medium was removed and the precipitated formazan crystals were dissolved in optical grade DMSO. The plates were read at 570 nm on a Victor 2 spectrofluorimeter.

### Hypoxia treatment and cell sorting

HeLa-GFP cells were seeded at 7.5 × 10^5^ cells/mL on a 150 mm sterile dish, allowed to set for 24 h. Cultures were submitted to HX for 4 days in Thermo Electron Corp. incubator. Populations were sorted in a Beckman Coulter MOFLO High Speed Cell Sorter, according to the size and GFP signal intensity as explained in the main text. After sorting, cells were seeded in 6-well plates at a cell density of 1.5 × 10^5^ cells/mL, treated or not with 5 μmol/L SVT016426 and placed for additional 6 days in HX. Cell population analysis was performed using a Beckman Coulter cytofluorometer.

### Doxorubicin treatment and cell sorting

HeLa-GFP cells were seeded at 7.5 × 10^5^ cells/mL on a 150 mm sterile dish, allowed to set for 24 h and treated with 0.25 μmol/L Doxo for additional 24 h. Populations were sorted as described above and treated for 24 h with 0.5 μmol/L Doxo. Cell population analysis was performed in a Beckman Coulter cytofluorometer.

### Transmission electron microscopy

HeLa cells were seeded at 3 × 10^4^ cells per chamber in a Lab-Tek chamber slides of 4 wells (Nalge Nunc International) and treated as indicated in each case. Then, the cells were fixed for 1 h in 3.5% glutaraldehyde at 37°C and postfixed for 1 h in 2% OsO_4_ at room temperature. Cellular staining was performed at 4°C for 2 h in 2% uranyl acetate in the dark. Finally, cells were rinsed in sodium phosphate buffer (0.1 mol/L, pH 7.2), dehydrated in ethanol, and infiltrated overnight in Araldite (Durcupan, Fluka). Following polymerization, embedded cultures were detached from the chamber slide and glued to Araldite blocks. Serial semi-thin (1.5 μm) sections were cut with an Ultracut UC-6 (Leica), mounted onto slides and stained with 1% toluidine blue. Selected semi-thin sections were glued (Super Glue, Loctite) to araldite blocks and detached from the glass slide by repeated freezing (in liquid nitrogen) and thawing. Ultrathin (0.07 μm) sections were prepared with the Ultracut and stained with lead citrate. Finally, photomicrographs were obtained under a transmission electron microscope (FEI Tecnai Spirit G2) using a digital camera (Morada, Soft Imaging System, Olympus).

### ATP measurements

HeLa cells were seeded at a density of 5 × 10^4^ cells/mL and treated as indicated. ATP measurement was performed in duplicate employing ATPLite kit (Perkin Elmer) according to manufacturer’s instructions. The ATP content was normalized per number of cells.

### Statistical analysis

Data were analysed using GraphPad software and statistical significance was assessed by means of two-tailed Student’s *t*-test (*P < 0.05). All data were expressed as the mean ± SD.

## ABBREVIATIONS

AIP: Apaf1 inhibitory protein
Apaf-1: apoptotic protease-activating factor 1
Cyt *c*: cytochrome c
Cyt *c*-GFP: fluorescent GFP-labelled Cyt *c*
dATP: deoxyadenosine triphosphate
Doxo: doxorubicin
HeLa-GFP: human cervix adenocarcinoma (HeLa) cells stably expressing Cyt c-GFP
LC3-I: microtubule-associated protein 1A/1B-light chain 3 alpha type I
LC3-II: microtubule-associated protein 1A/1B-light chain 3 alpha type II
MOMP: mitochondrial outer membrane permeabilization
TEM: transmission electron microscopy
zVAD-fmk: Z-Val-Ala-Asp(OMe)-fluoromethylketone
